# Interventions to reduce neonatal mortality from neonatal tetanus in low and middle income countries - a systematic review

**DOI:** 10.1186/1471-2458-13-322

**Published:** 2013-04-09

**Authors:** Adeel Ahmed Khan, Aysha Zahidie, Fauziah Rabbani

**Affiliations:** 1 Department of Community Health Sciences, The Aga Khan University, Karachi, Pakistan

**Keywords:** **“**Tetanus”, “LMIC”, “Interventions”

## Abstract

**Background:**

In 1988, WHO estimated around 787,000 newborns deaths due to neonatal tetanus. Despite few success stories majority of the Low and Middle Income Countries (LMICs) are still struggling to reduce neonatal mortality due to neonatal tetanus. We conducted a systematic review to understand the interventions that have had a substantial effect on reducing neonatal mortality rate due to neonatal tetanus in LMICs and come up with feasible recommendations for decreasing neonatal tetanus in the Pakistani setting.

**Methods:**

We systemically reviewed the published literature (Pubmed and Pubget databases) to identify appropriate interventions for reducing tetanus related neonatal mortality. A total of 26 out of 30 studies were shortlisted for preliminary screening after removing overlapping information. Key words used were “neonatal tetanus, neonatal mortality, tetanus toxoid women”. Of these twenty-six studies, 20 were excluded. The pre-defined exclusion criteria was (i) strategies and interventions to reduce mortality among neonates not described (ii) no abstract/author (4 studies) (iii) not freely accessible online (1 study) (iv) conducted in high income countries (2 studies) and (v) not directly related to neonatal tetanus mortality and tetanus toxoid immunization (5). Finally six studies which met the eligibility criteria were entered in the pre-designed data extraction form and five were selected for commentary as they were directly linked with neonatal tetanus reduction.

**Results:**

Interventions that were identified to reduce neonatal mortality in LMICs were: a) vaccination of women of child bearing age (married and unmarried both) with tetanus toxoid b) community based interventions i.e. tetanus toxoid immunization for all mothers; clean and skilled care at delivery; newborn resuscitation; exclusive breastfeeding; umbilical cord care and management of infections in newborns c) supplementary immunization (in addition to regular EPI program) d) safer delivery practices.

**Conclusion:**

The key intervention to reduce neonatal mortality from neonatal tetanus was found to be vaccination of pregnant women with tetanus toxoid. In the resource poor countries like Pakistan, this single intervention coupled with regular effective antenatal checkups, clean delivery practices and compliance with the “high- risk” approach can be effective in reducing neonatal tetanus.

## Background

Neonatal tetanus has been a major cause of neonatal mortality all over the world. Global evidence has revealed tetanus as the highest mortality contributor among children after measles in vaccine preventable diseases [1]. In 1988, WHO estimated around 787,000 newborns deaths due to tetanus with an estimated proportionate mortality rate due to neonatal tetanus as 6.7 per thousand live births, showing the high magnitude of tetanus in global neonatal mortality [2]. Based on these alarming statistics, the 42nd World Health Assembly (WHA) called for the elimination of maternal and neonatal tetanus by 1995 [2].

Elimination is defined as “having less than 1 case per thousand live births in every district of the country.” Since the rate of immunization was not high at that time the target to eliminate neonatal and maternal tetanus was postponed to 2005 [3]. In 2008, WHO estimated 92% global reduction in tetanus related mortality as compared to frightening figures of 1988 when 787,000 newborns were reported to die due to tetanus [2]. However it was again a cause of concern that majority of the reported incidence in 2008 was concentrated in poor or middle income countries [2,4].

Data from Pakistan suggests that under-five mortality rates have declined from 117 to 94 per 1000 live births during 1991–2007. However, the neonatal mortality rates have remained almost static from 56 to 54 per 1000 live births [5]. Comparing 1988 statistics to 2010, there has been only 50% incidence reduction in neonatal tetanus in Pakistan compared to a global reduction of 82% [2,6]. Moreover, tetanus continues to be described as a ‘silent killer’ in Low Income Countries (LICs) as many neonatal deaths from remote and difficult to access areas largely remain unreported. While in LMIC, traditional sources of information about the descriptive epidemiology of diseases, injuries, and risk factors are generally incomplete, fragmented, and of uncertain reliability and comparability, and verbal autopsy is the only method to assess burden of tetanus.

High Income Countries (HICs) such as (United States of America, England, Denmark) have long ago controlled tetanus related neonatal mortality as compared to LICs [7-9]. Also some Low and Middle Income Countries (LMICs) like Nepal, Turkey, and Bangladesh are on track to reduce neonatal mortality due to tetanus [3]. These countries have implemented effective intervention packages e.g. safe birth and postnatal care practices, better cord care, better tetanus toxoid coverage of pregnant women etc [10-12]. However, in spite of some success stories in countries like Nepal and Bangladesh majority of the LMICs are still struggling to reduce neonatal mortality due to neonatal tetanus [2]. According to latest WHO statistics of 2011, 38 countries of Africa and Asia have yet not been successful in elimination of neonatal tetanus and Pakistan is one of them [3]. It’s important to highlight here that each case of neonatal tetanus eventually represents multiple failures of health services to provide routine immunization, antenatal care, and clean delivery and cord care services in these countries.

This study therefore aims to conduct a systematic review to understand the interventions that have had a substantial effect on reducing neonatal mortality rates in LMICs to come up with feasible recommendations for decreasing neonatal mortality rate (NMR) due to neonatal tetanus in the Pakistani setting.

## Methods

We systemically reviewed the published literature to identify studies regarding interventions meant to reduce neonatal mortality. A comprehensive search using specific key terms was conducted. ‘Pub med’ and ‘Pub get’ were used as resource database. Search key terms were “neonatal tetanus, neonatal mortality, and tetanus toxoid women”. These terms were employed simultaneously in both search engines. We included all papers published in English language, having an abstract and describing interventions put in place to reduce neonatal mortality due to neonatal tetanus. The review period comprised of articles published from June 2006 to June 2011.

From ‘Pub med’ search we got 20 articles while ‘Pub get’ search furnished 10 articles. After going through all the articles and sorting out those which were common to both of the databases final search was comprised of 26 articles. Out of these, 20 studies were excluded which (i) did not have described the strategies and interventions to reduce mortality among neonates (8 studies) (ii) had no abstract/author (4 studies) (iii) not freely accessible online (1 study) (iv) studies conducted in developed countries (2 studies) and (v) studies that were not directly related to neonatal tetanus mortality and tetanus toxoid immunization (5 studies). Finally six studies which met the eligibility criteria were entered in the data extraction form.

### Data extraction

**Figure 1:**
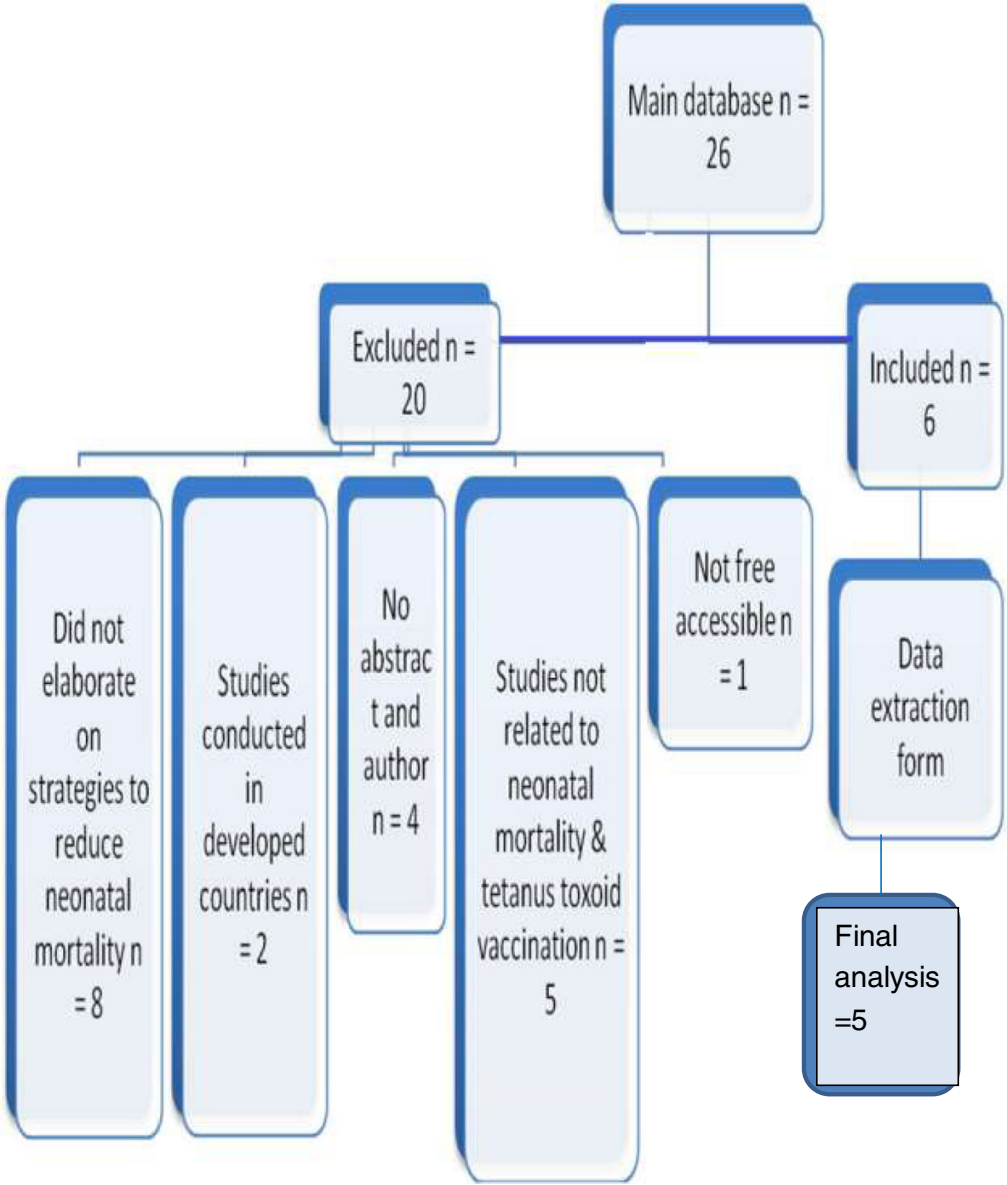
Flow chart for the synthesis of articles.

Information from the shortlisted 6 articles was enlisted into a predesigned data extraction form and five were selected. Of these five articles, two were systematic reviews, two were cross sectional studies and one was a review article. The one excluded from final analysis was on iron and folic acid supplementation to the pregnant women to improve chances of neonatal survival by improved immune status. However this study could not elaborate on specific reduction of neonatal mortality due to tetanus. Hence it was removed from our review (Figure 1).

## Results & discussion

In this section we have described the salient interventions for reducing neonatal mortality rate due to neonatal tetanus based on an analysis of these 6 studies (Table 1). Feasibility of implementation of each intervention for Pakistan has also been reviewed in the light of existing evidence.

**Table 1:** Articles related to interventions for reducing neonatal mortality from neonatal tetanus

**S. No.**	**Title of article**	**Name of journal and year of publication**	**Study design**	**Study site**	**Key findings**
**1.**	**Community-based intervention packages for reducing maternal and neonatal morbidity and mortality and improving neonatal outcomes (Review)**	The Cochrane Collaboration and published in The Cochrane Library	Systematic review (Cochrane)	India, Bangladesh, Pakistan, Gambia, Nepal, Indonesia	Significant reduction was observed in maternal morbidity, neonatal mortality, stillbirths and perinatal mortality as a consequence of implementation of community-based care packages.
			Review included 18 cluster-randomized/quasi-randomized trials		
		2010, Issue 11			
**2.**	**Tetanus toxoid immunization to reduce mortality from neonatal tetanus**	International Journal of Epidemiology 2010	Systematic review	India, Columbia	Immunization of pregnant women or women of childbearing age with at least two doses of tetanus toxoid was estimated to reduce mortality from neonatal tetanus by 94%.
**3.**	**Process of neonatal tetanus elimination in Nepal**	Journal of Public Health 2009	Cross sectional survey	Nepal	National tetanus toxoid supplemental immunization activities in 2000–2004, raised the proportion of children protected at birth against tetanus to above 80%.
**4.**	**Intra- and inter-household differences in antenatal care, delivery practices and postnatal care between last neonatal deaths and last surviving children in a peri-urban area of India**	J. Biosoc. Sci.2010	Comparative cross sectional	India	Higher percentage of tetanus toxoid immunization, safer delivery practices, postnatal care, higher maternal age and greater birth spacing were likely to reduce neonatal mortality.
**5.**	**Maternal and neonatal tetanus**	The Lancet 2007	Review paper	India, New Guinea, Columbia, Pakistan	The proposed packages of interventions include improved antenatal care, tetanus toxoid immunization of mothers, and promotion of hygienic delivery and postpartum cord-care, all of which will directly contribute to prevention of maternal and neonatal tetanus.

### Immunization of pregnant women or women of child bearing age with tetanus toxoid

Four out of six studies have emphasized immunization of pregnant women with tetanus toxoid as the most effective intervention.

First article included was a systematic review which was conducted with the objective of estimating the effect of tetanus toxoid immunization of pregnant women, or women of childbearing age on neonatal tetanus mortality [13]. In this review one randomized controlled trial (RCT) conducted in Columbia and one cohort study conducted in India were found to meet criteria for meta-analysis. The meta-analysis of this review revealed that immunization of pregnant women or women of childbearing age with at least two doses of tetanus toxoid is estimated to reduce mortality from neonatal tetanus by 94% (CI 80–98%). On the basis of reliable mortality data and large effect size the overall quality of the evidence was estimated to be moderate in this review. The main limitation of this review and the resulting effect estimate is the scarcity of high-quality trials. For our description, high quality review would be one based on at least 2 high-quality primary studies with consistent results while a moderate quality review would be based on at least 1 high-quality primary study and at least 2 primary studies with methodological limitations but consistent results.

The second study in Indonesia found that iron and folic acid supplementation for mothers together with tetanus toxoid vaccination had a protective effect in the neonatal period. In this study, pooled data on neonatal survival in singleton infants born in the 5 years before each of the Indonesian Demographic and Health Surveys of 1994, 1997 and 2002–2003 was analyzed. Multivariate Cox proportional hazards models were used to identify factors linked to early neonatal death. Hazard ratio was used as a measure of association in the study. With a large sample size of 40 576 infants it was found that the risk of early neonatal death was significantly reduced for infants of mothers who received either any form of antenatal care (hazard ratio, HR: 0.48; 95% confidence interval, CI: 0.31–0.73), any quantity of iron and folic acid (HR: 0.53; 95% CI: 0.36–0.77) or ≥ 2 tetanus toxoid injections (HR: 0.66; 95% CI: 0.48–0.92) [14].

The third study which was a narrative review showed the positive impact of tetanus toxoid vaccination in reducing neonatal mortality in LMICs (India, New Guinea and Columbia) [15].

In India, complete prenatal immunization with tetanus toxoid during pregnancy (two doses 1 month apart) was associated with 88% reduction in the risk of neonatal tetanus among the newborns [95% confidence interval CI 59% to 98%] [16]. In New Guinea, where the earliest trials were conducted to find out the efficacy of tetanus toxoid it was revealed that three doses of fluid tetanus toxoid (without adjuvant; equivalent to about two doses of aluminum-adsorbed tetanus toxoid) had an efficacy of 94% for the prevention neonatal tetanus mortality [17]. The trial conducted in Columbia demonstrated that there was no tetanus related mortality among the neonates born to the mothers who had received two or three doses of aluminum adsorbed tetanus toxoid within the previous 5 years [18].

The fourth study conducted in a peri-urban area of India reinforced that tetanus toxoid immunization during pregnancy protected against neonatal mortality. It showed that acceptance of tetanus toxoid during pregnancy was significantly higher among those women who have no history of neonatal death during the last pregnancy i.e. 53% as compared to 29% in those women with history of neonatal deaths [p < 0.001] [19].

Tetanus toxoid immunization is already recommended by WHO for pregnant women or women of childbearing age to prevent neonatal tetanus [2]. The delivery and acceptance of recommended vaccinations however is an enduring challenge in Pakistan as more than 50% of the districts are considered high risk for maternal or neonatal tetanus due to low tetanus toxoid coverage. In a study conducted in Khyber Pakhtunkhwa (KPK) province, 65% of women in urban areas were vaccinated [20]. The reasons cited for low immunization in the KPK were lack of awareness, low literacy, low accessibility and misconceptions regarding immunizations. These conditions were also significantly associated with poor socioeconomic conditions of the province. These macro issues need to be addressed urgently if any considerable progress towards the goal of maternal and neonatal tetanus elimination is to be achieved. Studies have shown that the visits of lady health workers were also significantly associated with the vaccination status of the married women [20,21]. Lady health workers should be effectively utilized in order to increase the vaccination coverage.

### Community based interventions for reducing neonatal mortality

One of our included studies was a Cochrane systematic review, addressing the community based interventions that were applied for reducing maternal and neonatal morbidity and mortality and improving neonatal outcomes [22]. Twelve studies conducted in LMICs of total sample size 136,425 concluded that community based interventions such as tetanus toxoid immunization to mothers; clean and skilled care at delivery; newborn resuscitation; exclusive breastfeeding; umbilical cord care and management of infections in newborns resulted in 24% reduction in neonatal mortality (average RR 0.76; 95% CI 0.68 to 0.84). In a subgroup analysis of the same review it was shown that four studies from India, Bangladesh and Pakistan, carried out an estimation of the impact separately for packages that built support and advocacy groups i.e. home visits along with community mobilization, showing significant reduction in average neonatal mortality by 21% (average RR 0.79; 95% CI 0.68 to 0.92). Also interventions like home-based neonatal care and sepsis management had substantial effect on reduction of neonatal mortality (average RR 0.43; 95% CI 0.27 to 0.69) in one study conducted in India (sample size 2089) [22].

This study illustrated that immunization of pregnant women does result in an impact on disease burden, but could not achieve levels that were sufficient to achieve neonatal tetanus elimination without combination of other community based interventions.

While the importance of facility-based services for maternal and newborn care cannot be denied, this study provides sufficient evidence to scale up community-based care through packages which can be delivered by community health workers.

Community based interventions are practical and feasible in the context of low income countries like Pakistan, India, Gambia etc [23]. There is evidence from other studies conducted in Pakistan showing that almost half of the newborn deaths due to infectious causes especially tetanus can be prevented by developing integrated community-based intervention packages complemented by developing and strengthening local health systems and working through peripheral lady health workers [24].

### The high risk approach and Tetanus toxoid supplemental immunization activities (SIA’S)

The narrative review included in our study pointed towards the effectiveness of adopting High Risk Approach for neonatal tetanus mortality reduction [15]. In areas where immunization fails to reach to a substantial proportion of pregnant women, tetanus toxoid SIAs may be required. This is known as the “high-risk approach”.

High risk areas were identified based on data from tetanus surveillance, proportions of deliveries by traditional birth attendants and proportion of women with at least two doses of tetanus toxoid. After selecting high risk areas, supplemental immunization activities were organized in these areas with the target of immunizing all women of child bearing age. Educational programs were also launched to educate the mothers and traditional birth attendants regarding safe delivery and clean cord practices. It was found to be an economical approach for the reduction of neonatal tetanus.

In Pakistan, it was shown that the cost of three rounds of tetanus toxoid vaccine was $117 per avoidable death and $3.61 per disability adjusted life years (DALY) averted [15]. In Egypt, successful implementation of these activities resulted in significant reduction of neonatal tetanus cases i.e. no. of cases reduced from 2000 cases per year to less than 100 per year during 1992 to 2002 [15].

Tetanus toxoid supplemental immunization activities have proved to be effective in reducing the tetanus related mortality and morbidity at country level. Tetanus elimination process in Nepal has shown that despite of having only 10% deliveries with skilled personnel, Nepal has been able to eliminate neonatal tetanus due to supplementary immunization activity (SIA) supported by UNICEF. Overall, more than 80% of the targeted women received at least two doses of tetanus toxoid and over 75% of women received three doses. This activity has contributed in achieving the target of tetanus elimination in Nepal in a short span of six years [25].

The Maternal and Neonatal Tetanus Elimination Initiative was launched by UNICEF, WHO and UNFPA in 1999, uplifting the goal of maternal and neonatal tetanus elimination as a major public health problem.

As far as situation in Pakistan is concerned, sub immunization campaigns for measles and polio are already being carried out throughout the country. However, the only comprehensive sub immunization campaign that was conducted for prevention and control of vaccine preventable diseases among vulnerable population was after the devastating floods of 2010 which affected around 20 million people across all five provinces of the country. This campaign targeted all under five children and the pregnant women in internally displaced people camps. Women were administered with one dose of tetanus toxoid. However the beneficial effect of this activity needs to be objectively evaluated in terms of beneficial outcome for reduction of maternal and neonatal tetanus [26].

### Antenatal care & safer delivery practices

In LMICs, deliveries in unsafe and unhygienic environment have been an issue for years. An Indian study conducted in a peri-urban setting of the country in 2010 showed that deliveries in health facility have remarkably low rate of neonatal mortality as compared to deliveries conducted at home by un-trained birth attendants. This study concluded that most of the neonatal deaths took place when the deliveries were assisted by untrained birth attendants (84%) [19].

Among the women with “no” neonatal deaths, percentage of involvement of untrained birth assistants was quite low (62.1%) as compare to women with neonatal deaths, where the percentage of untrained birth assistants was found to be 71% (*p* < 0.001). It was also found that during the birth of the last living child, the instrument used for cutting the umbilical cord was more likely to be sterilized (85%) than during the birth of the dead children (75%) and the difference was significant between two groups (*p* < 0.001) [19].

Antenatal care during pregnancy also has also been proved to be a significant factor for reduction of neonatal mortality. In the review conducted at peri-urban setting in India, it was revealed that the rate of antenatal checkups was 12% among pregnant women with last neonatal deaths than the rate of antenatal checkups of the pregnant women when last living children were born (41%) (p < 0.001) [19].

This study was conducted in a background when as a part of the National Rural Health Mission since 2005, every village in India has been provided with a trained female community health activist, Accredited Social Health Activist (ASHA) (National Rural Health Mission, 2009). In this study ASHA is supposed to create awareness about danger signs among pregnant women. She also mobilized the local community for health seeking behaviors and encouraged community for the utilization of existing health services. The introduction of ASHAs may have played a very important role in making women more aware of antenatal care, safe delivery practices and newborn care in the study area resulting in better health care among the younger mothers and newborn children in the last decade. Two more things can be ascertained from the results of this study. Older women with no neonatal deaths adopted safer delivery care and methods, while some women living in the same peri-urban area failed to do so, indicating inter-household differences in combating neonatal deaths. Secondly, older women with neonatal deaths had differences in their age of giving birth to the last live birth and last dead child. Most of the neonatal deaths took place when the women were young, and gradually with increasing age the chances of child survival increased. This individual difference in behavior can be attributed to adoption of safer delivery methods, with increasing age and experience of the same individual. The reduction in neonatal deaths during last few years can be described with respect to the strengthening of immunization practices, antenatal programs and safe delivery practices and this is again replicated in this study by a lower incidence of neonatal deaths in younger women. Antenatal care programs and safe delivery practices directly contribute to the reduction of neonatal tetanus by prevention of infection on one hand and enhancing tetanus immunization opportunities of the pregnant women on the other hand. The observed significant differences in antenatal care, delivery and postnatal care between women with neonatal deaths and women, who had no neonatal deaths, living in the vicinity of an urban city and having easy access to health care and safe delivery institutions, show the impact of care on the pregnancy outcome and the indicators of child health.

The results of the study can be applicable to the Pakistani context as well. In Pakistan, various vertical programs already focus on women health and Emergency Obstetric Care (EmOC) is the latest initiative among strategies to provide safe delivery care. However, steps need to be taken to improve people’s confidence in the quality of available services and health care providers should be trained to ensure clean, hygienic delivery practices as it is of vital importance to achieve global target of tetanus elimination [27].

## Conclusion

Tetanus induced neonatal mortality is still a widespread problem in LMICs. As various interventions were discussed in this review, the key intervention which remarkably lowered the neonatal mortality was the vaccination of pregnant women or women of child bearing age with tetanus toxoid. Along with this, regular antenatal checkups, safe and clean deliveries, iron and folic acid supplementations and high risk approach were also effective in reducing neonatal mortality. Community Based Behavior Change Management is a possible intervention to overcome the problem in LMICs. Strengthening of health systems towards simple cost effective interventions is a key to successfully eliminating tetanus in countries like Nepal and Bangladesh.

In Pakistan, there remains an unfinished agenda of tetanus elimination and findings from this review should be taken as a benchmark for setting future priorities in near-term efforts to reduce neonatal mortality due to tetanus.

## Competing interests

The authors declare that they have no competing interests.

## Authors’ contributions

AK conceived the study and supervised the article search. AK, AZ and FR drafted and revised the manuscript. All authors read and approved the final draft.

## Pre-publication history

The pre-publication history for this paper can be accessed here:

http://www.biomedcentral.com/1471-2458/13/322/prepub
